# Evaluation of some heavy metals in water and health implications for fish consumers of the Great Cairo Sector of the Nile River

**DOI:** 10.1038/s41598-025-95308-z

**Published:** 2025-04-12

**Authors:** Alaa I. Khedr, Hala E. Ghannam

**Affiliations:** 1 National Institute of Oceanography and Fisheries, NIOF, Cairo, Egypt; 2 National Institute of Oceanography and Fisheries, NIOF, Cairo, Egypt

**Keywords:** Great Cairo Sector, Nile River, Target Hazard Quotient (THQ), Health Risk Index, *O. niloticus*, Heavy metal pollution index., Ecology, Ecology, Environmental sciences

## Abstract

Rivers serve crucial functions in the worldwide hydrological cycle. The industrial revolution, climate change, and urban development generated diverse water contaminants. This work aimed to assess the regional and seasonal distribution of some heavy metals (HMs) in the hot spot sites along the Great Cairo Sector, Nile River during 2021–2022. In addition, two commercial fish species (*O. niloticus and C. gariepinus*) were selected for assessing heavy metal content and human health risk. The results of heavy metals in water varied within; (1–7), (45–85.5), (19–84), (148–376), and (65–170) µg/l for Cd, Cu, Pb, Mn, and Zn, respectively over the study period. The heavy metal pollution index (HPI) results categorized the water status as unsuitable for drinking and aquatic life, but ideal for irrigation purposes. Based on Metal Index (MI) values, all examined sites were significantly at risk of metal contamination (> 1) over all uses. In the two investigated species, The results of Cd, Cu, Pb, Mn, and Zn varied in the edible part of fish species within (0.2–0.28), (2.01–5.41), (0.21–1.11), (12.1-15.25), (20.91–32.52) mg/g ww, respectively, for *O. niloticus* and within (0.2–0.35), (3.12–6.5), (1.52–3.62), (15.01–17.72), (15.12–26.93) mg/g ww, respectively, for *C. gariepinus* over the study period. The total annual daily intake of HMs was estimated to be 0.03625 and 0.03725 mg/kg.bw.day from the human consumption of *O. niloticus* and *C. gariepinus*, respectively. The Target Hazard Quotient (THQ) ranked in the order of Cd > Pb > Mn > Zn > Cu for *O. niloticus* and in the order of Pb > Cd > Mn > Cu > Zn for *C. gariepinus* with values lower than 1 that reported non-carcinogenic risk for consumers from the ingestion of investigated HMs seperately. Moreover, the Health Hazard Index (HI) slightly exceeded the threshold value of 1 of *C. gariepinus*, classified as moderate risk levels for consumers. Conversely, HI values were below 1 for *O. niloticus*, suggesting no risk from this species’ consumption. This study recommended an assertive water-quality monitoring strategy to mitigate health-related outbreaks and disruptions in aquatic ecosystems. The supplied data will undeniably assert environmental policymakers to implement sustainable pollution management and remediation measures.

**Supplementary Information:**

The online version contains supplementary material available at 10.1038/s41598-025-95308-z.

## Introduction

Freshwater is vital for people’s existence, and its purity has become a worldwide concern^1^. The Nile River serves as Egypt´s essential source of fresh water, fulfilling practically all drinking and irrigation requirements^2^. The Nile water is affected by the hydrologic frameworks controlled by the stream´s canals, as well as land and water utilization, encompassing agricultural runoff, industrial discharges, municipal waste, and effluents from river vehicles^3^. The rise in industrial, agricultural, and recreational activities, along with inadequately designed drainage and sewage systems and the Ethiopian dam, poses a significant threat to both the amount and quality of Nile water^4^. Egypt´s annual allocation of the Nile River´s water is 55 billion cubic meters. Due to upstream dam developments on the Nile River, water consumption by adjacent nations, and population growth, Egypt’s Nile River has seen significant environmental alteration^5^. The Nile River supplies roughly 95% of the nation’s freshwater needs. Numerous branches, irrigation canals, and streams arise from the Nile, constituting an agricultural system that encompasses 31,000 km^6^. The Nile River serve mostly for drinking water, irrigation, industrial purposes, transportation, and fishing. Nonetheless, it is regarded as the principal receptor of wastewater and drainage produced by various activities^4^, resulting in substantial organic and inorganic waste, along with heavy metals, being discharged into the Nile and subsequently flowing into the Mediterranean Sea^7^. Heavy metals represent a compelling class of elements regarding aquatic pollution due to their detrimental impact on ecosystem balance, prolonged persistence, capacity for accumulation in water and sediments, and organism bioaccumulation^8^. Among these metals, cadmium (Cd) and lead (Pb) are potentially poisonous and lack any known biological function, but copper (Cu) and zinc (Zn) are vital^9^. However, excessive consumption of critical metals by aquatic species might lead to detrimental effects^10^. Elevated metal concentrations in aquatic systems can lead to significant water pollution issues, including possible health concerns for humans via the food chain^2^. Metal contamination in aquatic ecosystems is increasing at alarming rates, constituting a significant global issue. Metal contamination is often evaluated by measuring HMs levels in water, sediments, and biotic organisms^11^. It appears unsuitable to classify metals as fluxes in aquatic ecosystems just by water analysis^12^. Metal pollution in aquatic habitats poses a substantial risk to public health^13^. Prolonged exposure to metals is a significant health risk, potentially affecting all bodily organs, including respiratory ailments, neurological issues, carcinogenic effects, and osteoporosis^14^.

The deterioration of water’s purity is merely one detrimental consequence of water contamination, which is jeopardizing people’s health, ecological systems, financial growth, and society as a whole^15,16^. The potential for sustainability to maintain the integrity of the Nile’s water and its tourism appeal for the advantage of future generations must also be emphasized. Drinking water must adhere to various criteria and regulations, including World Health Organization (WHO), and Egyptian Water Quality Standard guidelines (EWQS), to guarantee that the water provided to the public is safe^5^.

Fish serve as bio-markers in the environment, providing valuable insights into the level of infection from HMs and the associated risks for human consumption^17^. The muscle tissue of fish is the primary portion ingested by customers. The muscle is recognized for its role in metal biotransformation and accumulation in fish^18^, which provides more significant insights into the pollution state than measuring their levels in water^19^. Excessive consumption of heavy metals impedes the functionality of biomolecules, including proteins and enzymes, by compromising their structures^20^. Fish is regarded as the primary and most economical source of protein in Egypt, with *O. niloticus* and *C. gariepinus* being the most frequently consumed species along the Nile River^21^. Estimation of the health risk exposed to humans through fish consumption is an important issue. Health risk assessments are based on assumptions that most chemicals with noncancer effects, exhibit a threshold respon^22^. The Target hazard quotient (THQ) has been employed for individual heavy metals contamination and the hazard index (HI) for combined cumulative effects. As Humans are often exposed to more than one pollutant and suffer combined or interactive effects, HI might overestimate the potential for noncancer health effects^23^. This is because the toxicological effects associated with exposure to multiple chemicals, often through different exposure pathways^24^.

Consequently, a thorough evaluation of the state of the surface and gaining expertise are imperative to guarantee proper and secure water utilization. Water quality monitoring establishes an empirical foundation for the sustainable management of water resources and assists governments in evaluating, regulating, and forecasting water pollution^25^. Employing water quality and heavy metal contamination indices constitutes a straightforward and efficient approach to evaluating water quality^26^. Assessing the human health risk from HMs intake via fish is a major issue. The purpose of this work is to (1) evaluate the potential sources of some HMs (Cd, Cu, Pb, Mn, and Zn) along the Great Cairo sector of the Nile River, (2) assess the extent of metal pollution using multiple indices [Pollution Index (PI), Heavy Metal Pollution Index (HPI), and Metal Index (MI)]; (3) analyze and compare the heavy metal content in the edible portions of two selected commercial fish species (*O. niloticus* and *C. gariepinus*), and (4) assess the bioaccumulation factor (BAF) and the associated health risk related to the fish intake by adults (70 years).

## Results and discussion

### Physiochemical parameters

Water temperature is a significant element that directly and indirectly impacts the water quality and aquatic life. It has an impact on aquatic species´ composition and the rate of chemical processes^27^. Water temperature fluctuated within (30–31.7), (24.1–26.9), (16.3–19.2), and (20.1–22.3) °C during summer, autumn, winter, and spring, respectively. One-way ANOVA analysis showed significant differences in water temperature values between seasons (*P* < 0.001) and sites (*P* < 0.05). The annual mean water temperature is significantly higher at site 3 than at the other sites, which may be attributed to the cooling system of the electric power station at Shubra. One crucial factor for assessing the water quality is its pH value. It relies on the balance between carbon dioxide and carbonate–bicarbonate. pH values varied from 7.5 at site 2 during winter to 8.5 at site 3 during summer. pH fluctuated significantly between the different seasons at *p* < 0.001 (One-Way ANOVA). The recent pH values were within the allowable criteria (6.5–9) for drinking water, recreation, agricultural, and aquatic life water use. Saad et al.^28^ reported the lowest pH values of 7.86 and 7.89 at El-Hawamdia and Helwan stations, respectively. Electrical Conductivity (EC) indicates the concentration of dissolved electrolyte ions in the water. However, significant increases in EC may be an indicator of polluting sources affecting the water. EC fluctuated between 239 µs/cm at site 3 during autumn and 642 µs/cm at site 1 during spring (Table S1 in the supplementary file). The annual mean values of EC were 439.83 ± 47.54, 295.5 ± 48.67, 368 ± 23.4, and 518.75 ± 97.65 µs/cm at sites 1, 2, 3, and 4 respectively. EC fluctuated significantly between seasons at *p* < 0.001. EC values were significantly higher at site 1 than S3 and S4 at *p* < 0.05 (Table S1). This may be attributed to high mineral salts discharged from the Iron and Steel Company at Helwan. Elsayed et al.^29^ recorded EC values varied in the ranges of 538–1176 and 371–516 µs/cm in the Rosetta and Damietta branches, respectively. Dissolved Oxygen (DO) is a critical variable that provides a healthy aquatic ecosystem. DO concentrations were higher than the acceptable level of 4 mg/l for aquatic life^30^; they were in the range of (6.04–6.63), (6.3–7.4), (7.3–8.5), and (6.4–7.1) mg/l during summer, autumn, winter, and spring, respectively, with significant differences between seasons (*P* < 0.05), Table 1. Site 4 recorded the highest DO concentration of 8.5 mg/l (Table S1).

**Fig. 1:**
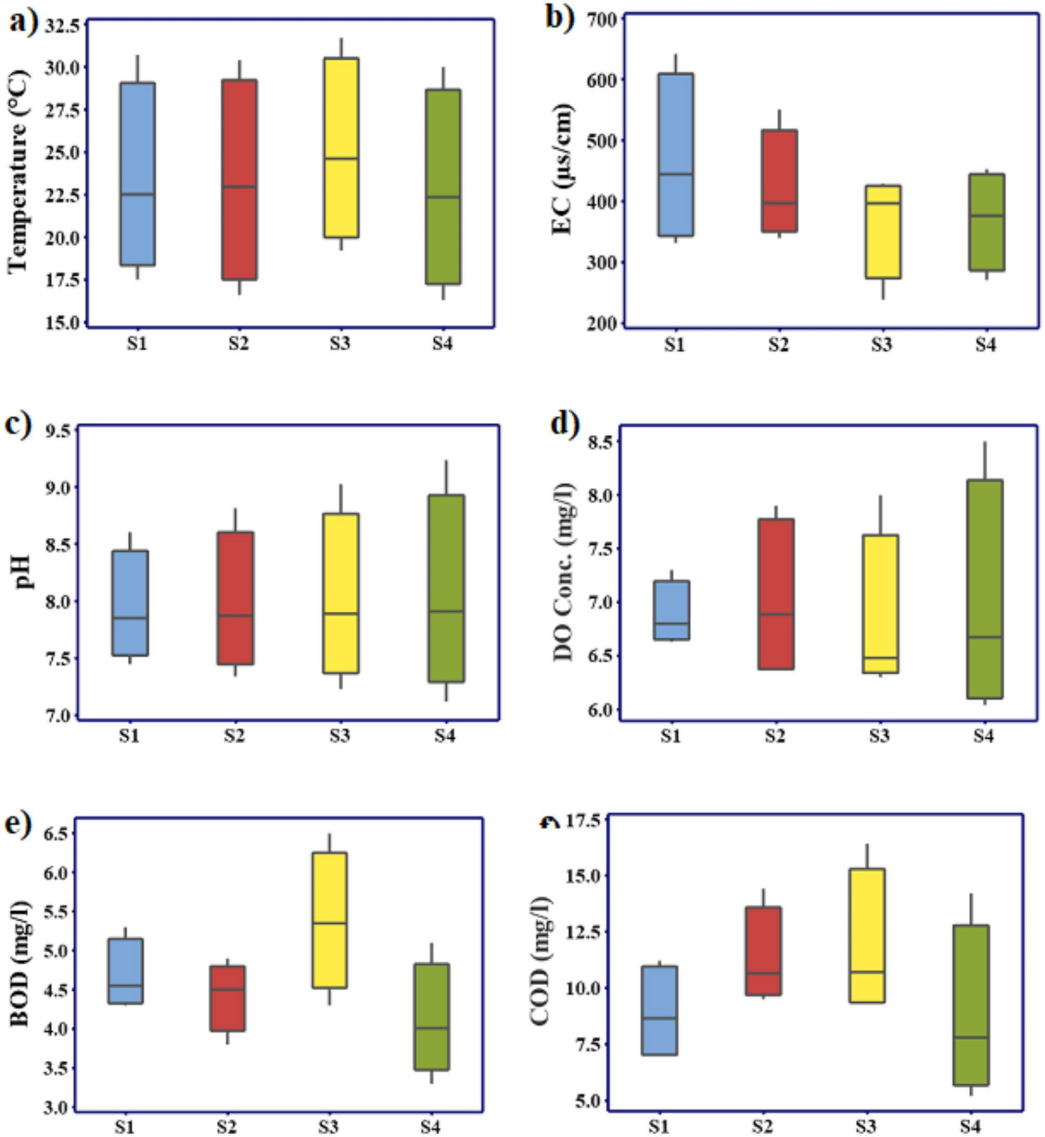
Box plot of (**a**) Water Temperature (°C), (**b**) EC (µS/cm), (**c**) pH, (**d**) DO (mg/l), (**e**) BOD (mg/l), and (**f**) COD (mg/l) concentrations in the Nile River water.

COD concentrations significantly varied between the sites within (7.4–7.8), (7.1–8.7), (7-8.5), and (7.5–8.3) mg/l; during summer, autumn, winter, and spring, respectively (ANOVA, *p* < 0.001). The maximum annual mean concentration of COD was 11.78 ± 3.3 mg/l at Site 3, affected by the drainage water of the Shubra electrical power station. However, Saad et al.^28^, Hussein et al.^31^, and Ali et al.^32^ recorded the maximum COD value at sites opposite to El-Hawamdia Company, affected by the effluent discharge from domestic sewage and agricultural discharge. BOD concentration ranged at the different sites within; (4–6.5), (4–5.2), (3.3–4.4), and (4.5–5.5) mg/l; during summer, autumn, winter, and spring, respectively, Fig. 1. All the sites showed BOD values within the acceptable limit (< 6 mg/l), except for site 3 during spring (Table S1). BOD recorded the lowest annual mean concentration at Site 4, El-Qanater El-Khayria, which agreed with the results of Ghannam^33^.

### Heavy metals

Anthropogenic and natural processes simultaneously contribute to the environmental abundance of HMs^34^. High concentrations of both necessary (Cu, Mn, Zn) and unnecessary HMs (Cd, Pb) can have detrimental effects on aquatic ecosystems, biodiversity, wildlife, and people^6^. Cd is a bioaccumulative, and lethal HM that lasts in the environment for a long period^35^. Cd fluctuated in the different sites within the range of (2–7), (2–4), (1–4), and (2–6) µg/l during summer, autumn, winter, and spring, respectively (Table S2). Cd showed high temporal and spatial variation at *p* < 0.05 and < 0.001, respectively. Cd exceeded the acceptable limits of 3 µg/l for drinking water criteria and irrigation at some sites. Moreover, it exceeded the guideline values accepted for aquatic life (0.72 µg/l) according to USEPA^30^. Cd is a cytotoxic metal to aquatic organisms that is regulated by biotic factors, such as organic content, hardness, and pH^4^. The present results reported higher Cd levels than that recorded of the Nile River (Nd-3.96), and (Nd-5) µg/l reported by Hussein et al.^31^, and Elnazer et al.^36^, respectively, and that recorded of Rosetta branch (0.81–2.3), Damietta branch (0.2-2) µg/l, Great Cairo (0.01–0.09) µg/l, and Ismailia Canal (Nd-2.94) µg/l, by Al Afify & Abdel-Satar^3^, Tayel et al.^37^, Omar et al.^38^, and Goher et al.^39^, respectively. Comparatively, Cd varied within the range recorded of the Nile Islands (0.7–9.3) µg/l by Abdel Satar et al.^40^; Bahr Yusuf Canal (1.86–8.25) µg/l by Hassouna et al.^41^; Nile River (0.2–8.1) µg/l by Abdel-Satar et al.^4^; and El Bahr El pharaony (Nd-7) µg/l by Goher et al.^42^.

Cu is an essential metal, but its high concentration may cause adverse biological impacts. Cu had concentrations between (56.25–85.5), (56-69.75), (39.3-56.25), and (45-65.25) µg/l during summer, autumn, winter, and spring, respectively, with a nonsignificant variation between seasons. The Cu concentrations were within the level allowed for drinking water (2000 µg/l) and irrigation (200 µg/l). Meanwhile, they greatly exceeded the acceptable limit (4 µg/l) for aquatic ecosystems. S2 showed the highest annual mean Cu concentrations followed by S1 and S3. The current Cu concentrations were higher than the previous results (6.8–73), (1.1–20.30), (14–72), (3.04–13.94), (4.1-46.33), (10–51), (0-13.6), (2–21.24), and (2.19–8.89) µg/l of Nile Islands, Nile River, Rosetta Branch, Bahr Yusuf Canal, Damietta branch, Nile River, El Bahr El pharaony, Ismailia Canal, and Great Cairo by Abdel-Satar et al.^40^, Hussein et al.^31^, Al Afify & Abdel-Satar^3^, Hassouna et al.^41^, Tayel et al.^37^, Abdel-Satar et al. 2017^4^, Goher et al.^42^, Goher et al.^39^, and Omar et al.^38^; respectively. However, they were lower than that recorded (Nd–170) µg/l of the Nile River by Elnazer et al.^36^.

Pb varied significantly between the different sites and seasons at *p* < 0.05. Pb had levels between (21–86), (19–48), (22–35), and (23–43) µg/l during summer, autumn, winter, and spring, respectively. The Pb concentrations greatly exceeded the guideline values for drinking water and aquatic life criteria, (Table 1). However, they were lower than the limit (5000 µg/l) reported for irrigation uses. Pb concentrations were significantly higher at S1 at *p* < 0.05. This study reported lower Zn levels than that recorded in the Nile River (163–402) µg/l by Elnazar et al.^36^ but higher than that recorded in the Nile Islands (3.5–66.5) µg/l, Nile.

**Table 1:** Water quality parameters and HMs levels (µg/l) in the present study in relation to the established allowable limits for drinking water, irrigation, and aquatic life.

	Summer	Autumn	Winter	Spring	Drinking Water		Irrigation	Aquatic Life
					EWQS^43^	WHO^44^	FAO ^45^	USEPA ^30^
Temperature (°C)	(30-31.7)^a^ 30.70 ± 0.73	(24.1-26.92)^b^ (25.33 ± 1.24)	(16.3–19.2)^d^ 17.40 ± 1.30	(20.1–22.3)^c^ 20.88 ± 1.01		12–25		8–28
pH	(7.75–7.81)^a^ 7.78 ± 0.03	(7.12–7.45)^c^ 7.29 ± 0.14	(8.6–9.23)^b^ 8.92 ± 0.27	(7.95–8.01)^b^ 7.98 ± 0.03	6.5–8.5	6.5–8.5	8.5	
EC (µs/cm)	(413–511)^a, b^ 439.75 ± 47.59	(239–340)^c^ 295.5 ± 48.67	(333–379)^b, c^ 368 ± 23.40	(429–642)^a^ 518.75 ± 97.65	2000		3000	
DO (mg/l)	(6.04–6.63)^b^ 6.34 ± 0.24	(6.29–7.4) ^b^ 6.77 ± 0.49	(7.3–8.5)^a^ 7.93 ± 0.49	(6.38–7.06) ^b^ 6.65 ± 0.31	6			5.5
BOD (mg/l)	(4.02–6.5)^a^ 5.03 ± 1.05	(4-5.2)^a, b^ 4.50 ± 0.51	(3.3–4.4)^b^ 3.95 ± 0.51	(4.5–5.5)^a^ 5.10 ± 0.43	3			
COD (mg/l)	(11.2–16.4)^a^ 14.05 ± 2.14	(5.2–11.9)^b^ 8.60 ± 3.01	(7-9.5)^b^ 8.28 ± 1.42	(8.5–11.1)^b^ 9.78 ± 1.12	10	10		
Cd (µg/l)	(2–7)^a^ 4.5 ± 2.08	(2–4)^a, b^ 3 ± 0.82	(1–4)^b^ 2.5 ± 1.29	(2–6)^a, b^ 3.75 ± 1.71	3	3	10	0.72
Cu (µg/l)	(56.2–85.5)^a^ 67.5 ± 12.86	(56.3–69.8)^a^ 63 ± 5.81	(45-56.3)^a^ 51 ± 4.75	(45-56.2)^a^ 51.19 ± 4.99	2000	2000	200	4
Pb (µg/l)	(21–86)^a^ 64 ± 30.19	(19–48)^b^ 32.75 ± 14.41	(22–35)^b^ 29.5 ± 5.57	(23–43)^b^ 30.75 ± 8.66	1	10	5000	2.5
Mn (µg/l)	(316–352)^a^ 333 ± 17.70	(176–340)^a, b^ 280 ± 74.69	(344–376)^a^ 365 ± 14.38	(148–264)^b^ 211 ± 48.15	100	100	200	100
Zn (µg/l)	(110–170)^a^ 148.75 ± 26.58	(65–110)^c^ 90.00 ± 19.58	(70–125)^b, c^ 102.50 ± 23.98	(95–130)^b^ 113.75 ± 16.52	500	3000	2000	120

Different letters refer to significant differences.

River (6.8–42.80) µg/l, Rosetta Branch (9.3–67.9) µg/l, Bahr Yusuf Canal (17.62–49.62) µg/l, Nile River (5–50.4) µg/l, El Bahr El pharaony (0.2–31.8) µg/l, Ismailia Canal (10.55–34.01) µg/l, Great Cairo (0.03–2.07) µg/l by Abdel-Satar et al.^40^; Hussein et al.^31^; Al Afify & Abdel-Satar^3^; Hassouna et al.^41^; Abdel-Satar et al.^4^; Goher et al.^42^; Goher et al.^39^; and Omar et al.^38^; respectively. Mn varied significantly between seasons at (*P* < 0.05) and fluctuated within (316–352), (176–344), (344–376), and (148–264) µg/l during summer, autumn, winter, and spring, respectively. Zn showed significant temporal and spatial variations at *p* < 0.001. High Mn concentrations were reported in this study compared with some previous results of (21.8–247.7), (14.5–62.30), (40–220), (23.29–69.35), (7.3-138.4), (30–298), (0.36–4.45) µg/l by Abdel-.

**Fig. 2:**
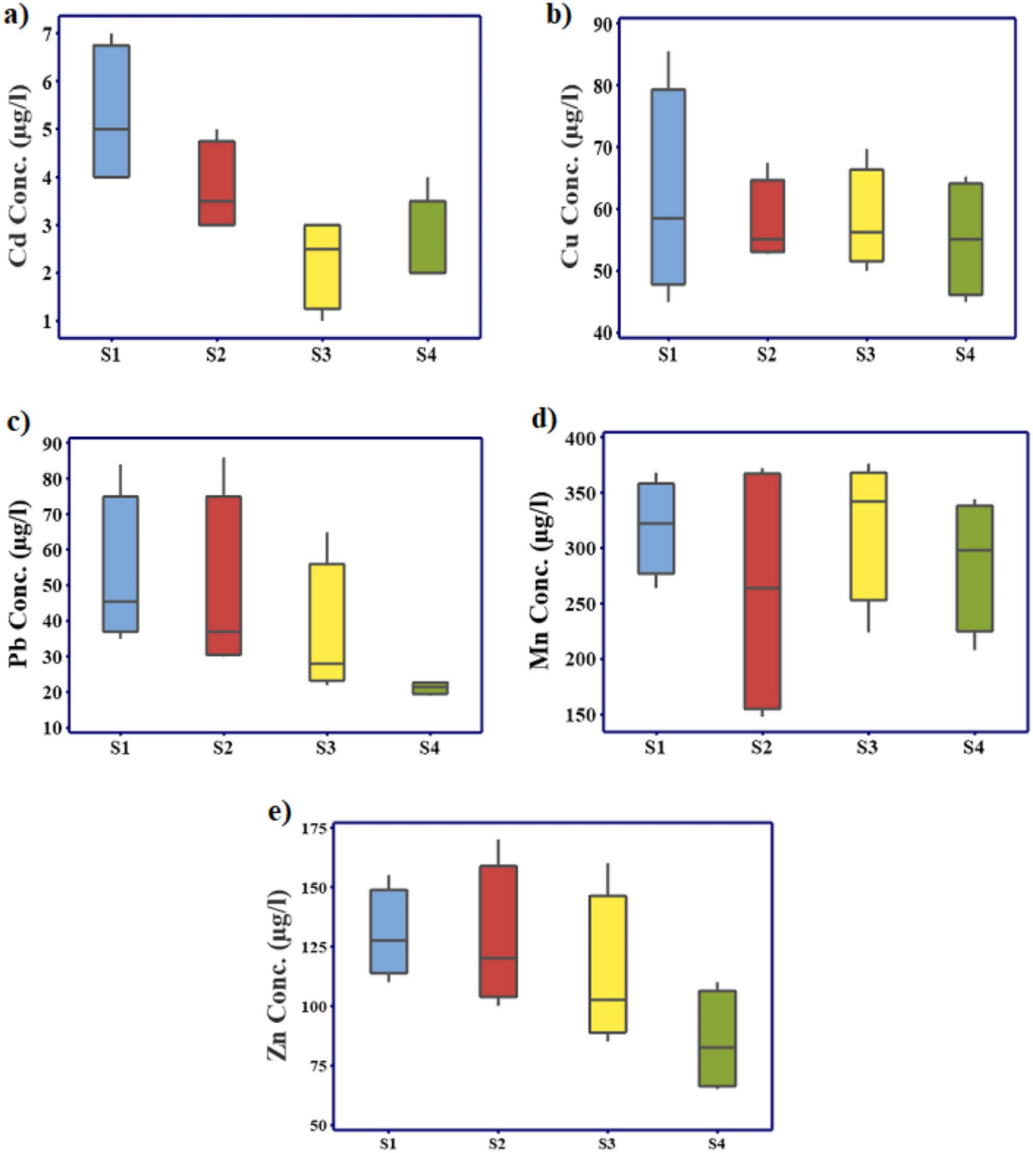
Box plot of (**a**) Cd, (**b**) Cu, (**c**) Pb, (**d**) Mn, and (**e**) Zn concentrations in the Nile River water.

Satar et al.^40^, Hussein et al.^31^, Al Afify & Abdel-Satar^3^, Hassouna et al.^41^, Tayel et al.^37^, Abdel-Satar et al.^4^, Omar et al.^38^; respectively, but still lower than that reported of Ismailia Canal (20–483.4) µg/l, and El bahr El pharaony (1.6–720) µg/l by Goher et al.^39,42^, respectively. Zn varied significantly within (110–170), (65–110), (70–125), and (95–130) µg/l during summer, autumn, winter, and spring, respectively, Fig. (2). S4 was significantly lower than the other sites (*p* < 0.001, One-Way ANOVA) which may be attributed to its farness from the pollution sources. The maximum annual mean concentrations of Cd, Cu, Pb, and Zn were 4.85, 61.88, 52.5, and 130 µg/l, respectively at Site 1, affected by the drainage water from Iron and Steel Company (Table S2). On the other hand, Mn showed a higher annual mean concentration (321 µg/l) at site 3 affected by the cooling water discharged from the Shubra Electrical Power Station (Table S2). Zn concentrations were slightly greater than that reported of Nile Islands (16–140 µg/l) by Abdel Satar et al.^40^ and Rosetta branch (28.55–117.36 µg/l) by Al Afify & Abdel-Satar^3^ but lower than that reported of the Nile River (50–700 µg/l) by Elnazar et al.^36^ and El Bahr El pharaony (3–222 µg/l) by Goher et al.^42^. The annual mean concentration of HMs decreased in the rank; Mn > Zn > Pb > Cu > Cd over the study period. Hashem et al.^46^ ranked the HMs concentration in Nile water in the order of Zn > Mn > Cu > Pb and referred to the highest pollution sites affected by the El-Rahawy drain during different seasons. A comparison of the current metal concentrations with other rivers around the world was reported in Table (2). The current Cu, Pb, Mn, and Zn concentrations in the Great Cairo sector were much less than that reported of Halda River, Bangladesh by Dye et al.,^47^ although Cd showed similar concentrations. In addition, the results of Cd and Pb concentrations were lower in compared with the Pasig River, Philippines reported by Paronda^48^. On the other hand, this study evaluated higher Cd and Cu concentrations in contrary to the Pearl River, China, and the Orontes River, Turkey reported by Jiao^49^ and Kilic & Can^50^.

**Table 2:** A comparison of the HMs results in the Nile River water with other previous studies in Egypt and other countries.

Study Area	Cd	Cu	Pb	Mn	Zn	Ref.
Great Cairo, Nile River, Egypt	1–7	45–85.5	19–84	148–376	65–170	Present Study
Great Cairo, Nile River, Egypt	0.01–0.09	2.19–8.89	0.03–2.07	0.36–4.45	20.48–55.81	38
Nile River, Egypt	0.2–8.1	10–51	5–50.4	30–298	10–108	4
Nile River, Egypt	Nd–3.69	1.1–20.30	6.8–42.80	14.5–62.30	9.90–68.20	31
Nile River, Egypt	Nd–5	Nd–170	163–402	–	50–700	36
Nile River, Rosetta Branch, Egypt	0.81–2.3	14–72	9.3–67.9	40–220	21.1–133	3
Damietta branch, Nile River, Egypt	0.2–2	4.1–46.33	–	7.3–138.4	28.55–117.36	37
Nile Islands, Egypt	0.7–9.3	6.8–73	3.5–66.5	21.8–247.7	16–140	40
Ismailia Canal, Nile River, Egypt	Nd–2.94	2–21.24	10.55–34.01	20–483.4	1.4–127.9	39
Bahr Yusuf Canal, Egypt	1.86–8.25	3.04–13.94	17.62–49.62	23.29–69.35	16.03–42.18	41
El Bahr El pharaony, Nile River, Egypt	Nd-7	0–13.6	0.2–31.8	1.6–720	3–222	42
Halda River, Bangladesh	1–8	–	700–2600	175–1185	399–941	47
Pasig River, Philippines	Nd-51	Nd-63	Nd-628	–	–	48
Pearl River, China	0.05–0.75	3–5	0.06–1.09	–	17–61	49
Orontes River, Turkey	Nd-0.16	2.01–20.9	0.3–3.3	13–1555	1–87	50

**Fig. 3:**
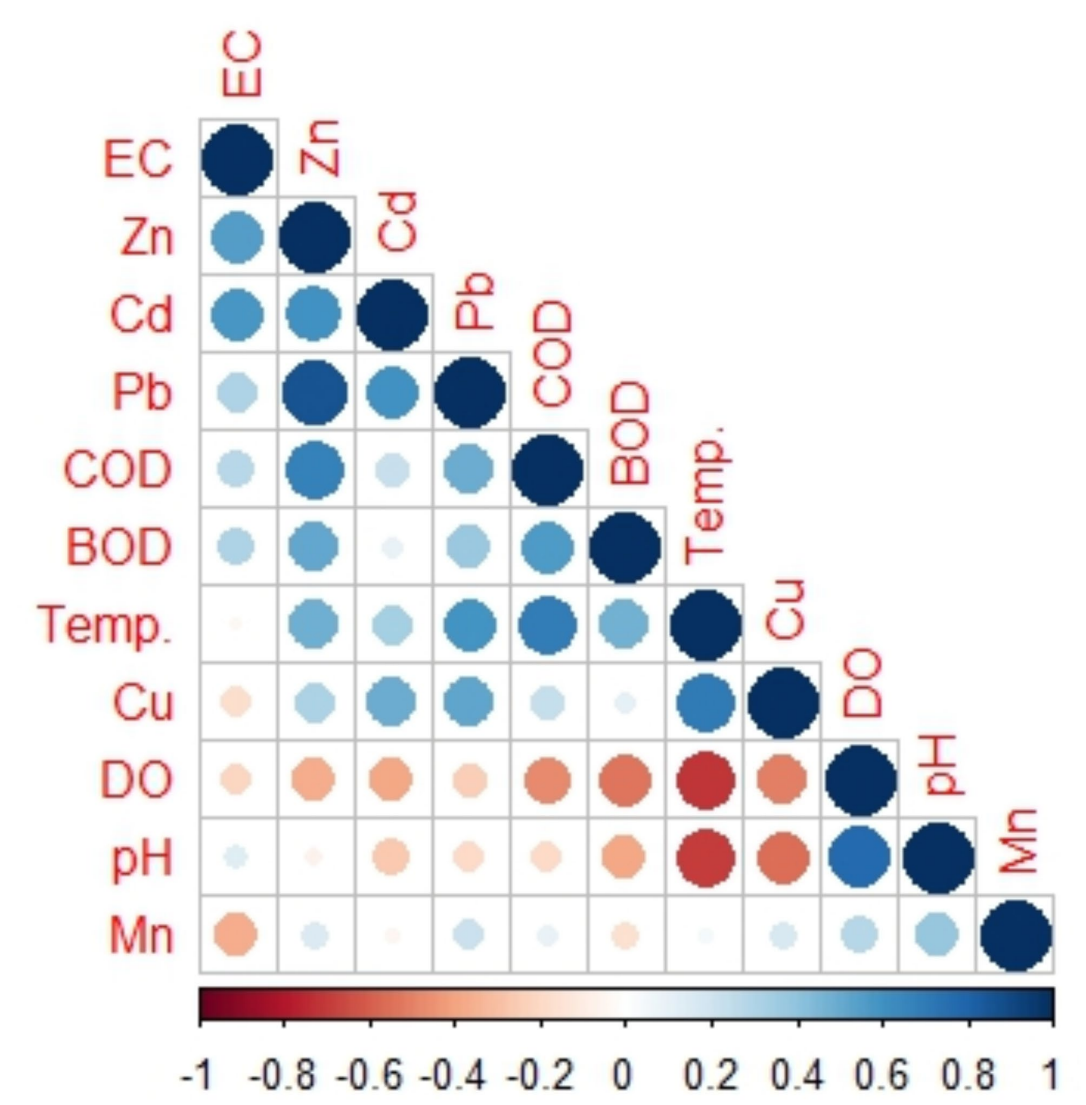
A correlogram illustrating the relationships between the variables under investigation in water samples. The circles’ color and size correspond to the correlation coefficients. Red color presents negative correlations while blue refers to positive correlations with different shades. Large-sized circles refer to a strong correlation and small- sized circles present a weak correlation. Correlations with *p*-value greater than 0.05 are deemed insignificant and are indicated by an empty white space.

The results of the Pearson correlation coefficient showed a strong negative correlation between DO and temperature (*r* = −0.73), which reflects low DO solubility at high water temperature values during summer (Fig. 3). Zn was positively correlated with EC (*r* = 0.51), COD (*r* = 0.69), BOD (*r* = 0.54), Cd (*r* = 0.52), and Pb (*r* = 0.85) but negatively correlated with DO (*r*=−0.35). The positive correlation reflects the potential sources of Cd, Pb, and Zn from the wastewater drains coupled with the increase of organic matter induced by COD and BOD concentrations and hence a rise in EC values and a decrease in DO consumed by high organic matter discharged. Moreover, Cu was negatively correlated with pH and DO (*r*= −0.69 and − 0.61), respectively. This reflects its source from the wastewater drains. Cu, Pb, and Zn were positively correlated with water temperature (*r* = 0.68, 0.50, 0.41), which reflects their higher levels during summer than the other seasons. That may be attributed to the liberation of metals from bottom sediment at high temperatures during the fermentation process^51^. This assumption agreed with the findings outlined by Goher et al.,^39,42^; Bahnasawy et al.^52^, Nwabueze and Oghenevwairhe^53^, Ibrahim and Omar^54^. On the other hand, Mn showed an elevated seasonal mean during winter, which may be the result of water-carrying soil from neighboring coasts, given the abundance of Mn in the earth´s outermost layers^55^.

### Pollution index

The PI is determined by the concentration of each metal, which allows us to predict the single effect of the pollution degree of each HM. Table 3 shows the PI values and categories of Cd, Cu, Pb, Mn, and Zn in the Nile River water based on standard values for drinking, irrigation, and aquatic life^30,43–45^. The PI values of Cd, Cu, Pb, Mn, Zn varied within; 0.53–1.3, 0.021–0.029, 1.49–4.55, 2-2.26, 0.02–0.03 according to EWQS^43^ drinking water criteria and within 0.53 - 1.34, 0.021–0.029, 14.92–45.54, 2-2.26, 0.13–0.20 according to WHO^44^ drinking water criteria. Cu and Zn showed PI values < 1 that showed a negligible effect on drinking water according to EWQS^43^ and WHO^44^ drinking water criteria. Cd had no effect on all sites except site 1 which was slightly affected according to EWQS^43^ and WHO^44^ drinking water criteria. Otherwise, Mn was moderately affected at all sites according to EWQS^43^ and WHO^44^ drinking water criteria. Pb varied from slightly affected at S4 (El Qanater) to strongly affected at the other sites according to EWQS^43^. Furthermore, Pb was seriously affected at all sites according to WHO^44^ drinking water criteria. For irrigation utilization, Cd, Cu, Pb, Mn, and Zn showed PI values of 0.16–0.4, 0.2–0.24, 0.003–0.009, 1–1.13, and 0.03–0.05, respectively according to FAO^45^. Cd, Cu, Pb, and Zn had PI values < 1 at all sites, exhibited no pollution effect, while Mn was slightly affected at all sites for irrigation uses. The PI values of Cd, Cu, Pb, Mn, and Zn for aquatic life varied within  2.2 - 5.6, 9.91–12.08, 5.97–18.22, 2-2.26, and 0.53–0.82, respectively, at the different sites according to USEPA^30^. Cd varied from moderately affected at S3 to strongly affected at S2 and S4, then seriously affected at S1. Furthermore, Cu and Pb showed PI values > 5, indicating a serious effect on the aquatic ecosystems at all sites according to USEPA^30^ criteria guidelines. Zn was slightly affected in the aquatic ecosystems at all sites, while Mn was moderately affected at all sites. Goher et al.^39^ documented a serious effect of Zn along the Ismailia Canal based on drinking and aquatic life standards while indicating slight to severe pollution by Pb consequences at all stations for drinking and aquatic life use.

### Metal index (MI)

**Fig. 4:**
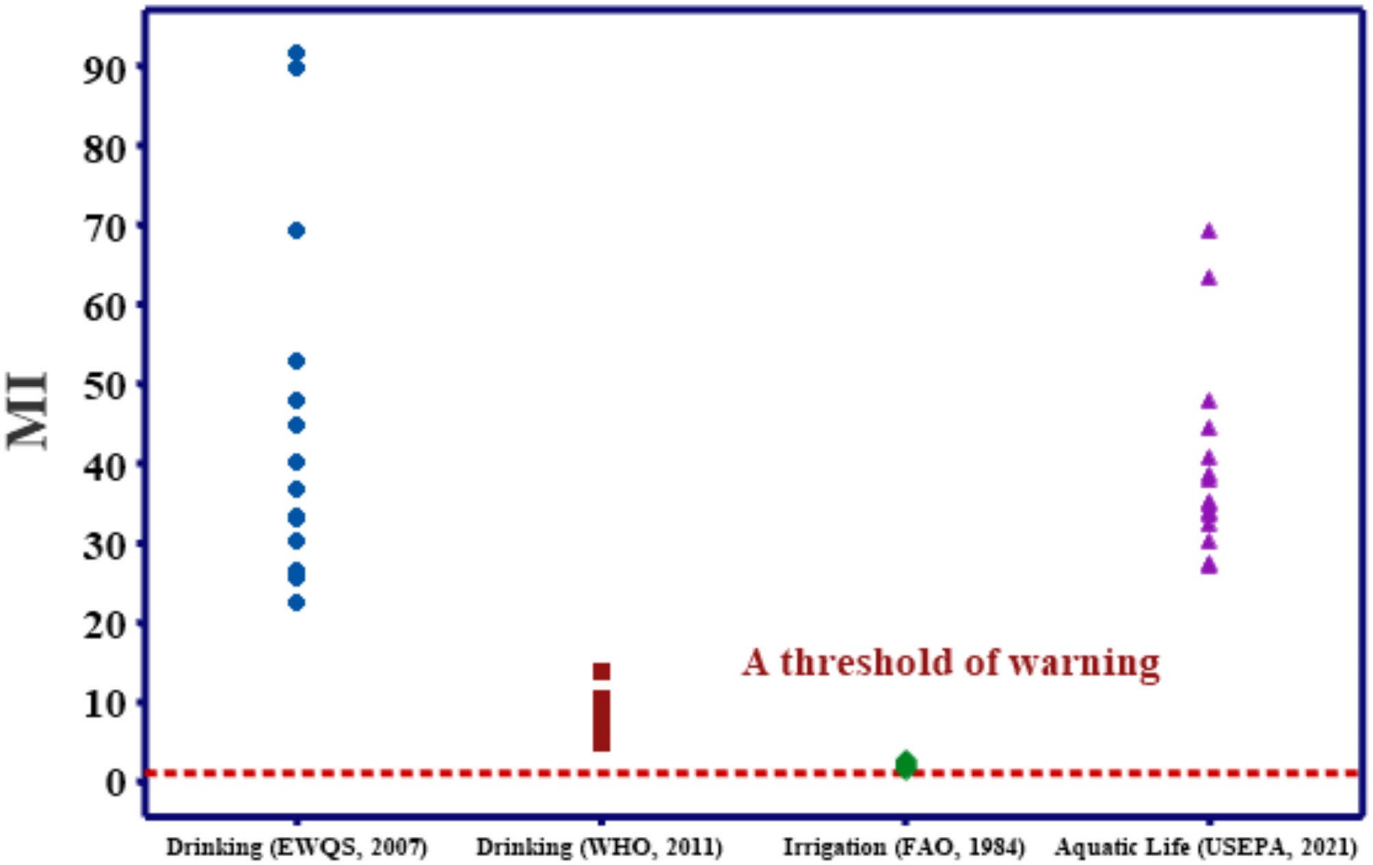
Individual plot of the MI values in the Nile River water.

A further indicator for determining the HMs contamination in the Nile River water for various uses is the Metal Index (MI). It takes into account all the measured HMs. MI values ranged within 22.59 -91.56 and 5.38–13.99 in the different seasons for drinking water according to EWQS^43^ and WHO^44^, respectively and 1.48–2.8 for irrigation according to FAO^45^ and 26.85–69.15 for aquatic life utilizations according to USEPA^30^. As MI values > 1, metal contamination poses a major risk to all of the sites under investigation for all purposes, Fig. 4. This may be due to the release of municipal, industrial, and irrigation waste products discharged into the surface river water^56^. Site 1 was the most contaminated site over the study period by the investigated HMs. Site 1 is directly affected by the drainage water from the iron and Steel Company. This effluent carries huge quantities of different metals. According to Goher et al.^39^, MI was greater than 1 along the Ismailia Canal and Nile River at all the sites and posed a major HMs danger for drinking water and aquatic life use. Moreover, Hussein et al.^31^ recorded MI ≥ 1 at all the selected sites along the Nile River that are extremely endangered by metal contamination for use in drinking water and aquatic life use. Abdel Satar et al. reported MI values in the Nile water exceeding the critical limit for drinking water according to EWQS Guidelines, indicating great contamination by HMs. Hassouna et al. recorded MI values in the Bahr Yusuf Canal exceeding the acceptable limit for aquatic life and pointed out the great risk exposed to aquatic organisms living there. Al-Afify et al.  reported that some metals in the Nile water surpassed the adaptability for aquatic life before and after the pollution source. Previous researchers linked the industries and agrochemicals as the primary sources of HMs in the Nile River water.

### Heavy metal pollution index (HPI)

Table 4 represents the HPI values of the Nile River. HPI exceeded 100 during the different seasons for drinking water according to EWQS^43^ and WHO^44^, and for the protection of aquatic life according to USEPA^30^. All sites were classified as extremely contaminated. On the other hand, HPI for irrigation fluctuated between 18 and 72, classified as low contaminated, and hence was excellent and suitable for irrigation use according to FAO^45^ (Table 4). The HPI results classified the water status of the investigated sites as undesirable for drinking and aquatic life, but it is excellent for irrigation utilizations.

**Table 3:** PI values of the investigated HMs in the Nile River water.

Site	Drinking ^43^		Drinking ^44^		Irrigation ^45^		Aquatic life ^30^	
Cd	PI	Effect	PI	Effect	PI	Effect	PI	Effect
S1	1.34	Slight	1.34	Slight	0.40	No	5.60	Serious
S2	0.97	No	0.97	No	0.29	No	4.05	Strong
S3	0.53	No	0.53	No	0.16	No	2.20	Moderate
S4	0.75	No	0.75	No	0.22	No	3.11	Strong
Cu								
S1	0.02	No	0.02	No	0.24	No	12.08	Serious
S2	0.02	No	0.02	No	0.21	No	10.71	Serious
S3	0.02	No	0.02	No	0.21	No	10.73	Serious
S4	0.02	No	0.02	No	0.20	No	9.91	Serious
Pb								
S1	4.55	Strong	45.50	Serious	0.009	No	18.20	Serious
S2	4.55	Strong	45.54	Serious	0.009	No	18.22	Serious
S3	3.43	Strong	34.31	Serious	0.007	No	13.72	Serious
S4	1.49	Slight	14.92	Serious	0.003	No	5.97	Serious
Mn								
S1	2.26	Moderate	2.26	Moderate	1.13	Slight	2.26	Moderate
S2	2.00	Moderate	2.00	Moderate	1.00	Slight	2.00	Moderate
S3	2.19	Moderate	2.19	Moderate	1.09	Slight	2.19	Moderate
S4	2.01	Moderate	2.01	Moderate	1.00	Slight	2.01	Moderate
Zn								
S1	0.03	No	0.19	No	0.05	No	0.79	Slight
S2	0.03	No	0.20	No	0.05	No	0.82	Slight
S3	0.03	No	0.18	No	0.05	No	0.75	Slight
S4	0.02	No	0.13	No	0.03	No	0.53	Slight

### HMs in fish

Aquatic organisms are impacted by HM contamination at the cellular level, leading to ecological discrepancies that may threaten the food chain. Fish are thought to be one of the best indicators of HMs toxicity in the aquatic environment^59^. Cd concentrations varied in the edible part of fish between (0.22 - 0.28) mg/g ww for *O. niloticus* and between (0.2–0.35) mg/g ww for *C. gariepinus*. These concentrations exceeded the permissible levels of (0.2, 0.1, 0.2, and 0.05) set by ANZECC/ARMCANZ^60^, EU^61^, MAFF^62^, and EC^63^, respectively. However, they were still lower than the FAO^64^ guideline value of 0.5 mg/g. The recent Cd concentrations were within the level recorded of *Mango tilapia* (Nd-0.3) mg/g ww, *Nile tilapia* (Nd-2) mg/g ww, and *Tilapia Zillii* (Nd-1.1) mg/g ww in the Rayahs, Nile River by Ghannam et al.^65^, but less than that recorded of *O. niloticus* (2.6–14.7) mg/g ww in the Damietta branch by Tayel et al.^37^. Cu concentrations varied between 3.12 and 6.5 mg/g ww in *C. gariepinus* and between 2.01 and 5.41 mg/g ww in *O. niloticus*. These concentrations were lower than the guideline values of (30, 30, 30, 10, 20 mg/g) set by FAO^66^, FAO/WHO^64^; WHO^67^; EU^61^, and MAFF^62^ respectively. In comparison, the current results of Cu were similar to those recorded of *O. niloticus* and *C. gariepinus* at the Great Cairo sector, Nile River by El Haddad et al.,^68^. Pb concentration fluctuated between (0.21–1.11) and (1.52–3.62) mg/g ww in the edible part of *O. niloticus* and *C. gariepinus*, respectively. The annual mean concentration of Pb in *O. niloticus* (0.638 ± 0.38 mg/g) exceeded the guideline values of (0.5, 0.5, 0.2, and 0.1) reported by FAO^3^, FAO/WHO^64^, EU^61^, and EC^63^, respectively. But it was still below the 2 mg/g allowable limits set by ANZECC/ARMCANZ^60^ and MAFF^65^. On the other hand, the annual mean concentration of Pb in *C. gariepinus* (2.665 ± 0.97 mg/g ww) exceeded all the stipulated permissible limits, (Table 5). Pb is known to be detrimental to human health regardless of the exposure rate, causing neurotoxicity and nephrotoxicity rapid behavioral and metabolic disorders, and growth retardation in humans^22^. The current Pb concentrations exceeded those documented of *O. niloticus* and *C. gariepinus*, in the Great Cairo, Nile River by El-Haddad et al.^68^. Mn concentrations varied within (12.1–15.25) and (15.01–17.72) mg/g ww in *O. niloticus* and *C. gariepinus*, respectively. These concentrations exceeded the WHO^67^ guideline value of 1 mg/g; nonetheless, they were below the permitted thresholds of 20 mg/g set by Dural et al.^69^. Zn concentrations fluctuated between 20.91 and 32.51 mg/g ww in *O. niloticus* and between 15.12 and 26.92 mg/g ww in *C. gariepinus* in the different seasons. The annual mean concentration of Zn was 27.29 ± 4.81 and 21.63 ± 5.14 for O. *niloticus* and *C. gariepinus*, respectively. It was lower than the permissible level allowed of (30, 40, 100) mg/g by FAO^66^, FAO/WHO^64^, and WHO^67^; respectively. This study inferred higher Zn levels than that recorded of Oncorhynchus mykiss trout (6.2–11.2 mg/g) in the North and Southeast Rivers, Iran, by Khammar et al.^13^.

Elevated concentrations of critical metals (e.g., Cu, Mn, and Zn) in the fish muscles may lead to diarrhea, abdominal cramps, tremors, anemia, alopecia, and facial muscle spasms^22^. Moreover, nonessential elements like Pb and Cd, irrespective of their amounts pose significant dangers, and can induce acute toxicity to human biological systems^22.^ Prolonged exposure to these elements from seafood sources may induce mutagenic, teratogenic, and carcinogenic health effects. This encompasses neurological and nervous system impairment, cardiovascular, renal, and liver failure, cancers, vascular trauma, telangiectasias, hormonal and endocrine dysfunction, infertility, digestive gland damage, and other systems, depending on the extent and pattern of uptake and the exposure route^22,23^.

The toxicity of HMs encompasses numerous factors. These HMs generate free radicals that lead to the oxidative degradation of biomolecules (DNA, proteins, and lipids)^70^. They can specifically target and relate to DNA strands to produce apotosis, tumor growth, and alterations in the cell cycle. They impede metabolic activities, tissue repair, and cellular and organ detoxification^71^. However, each metal possesses its unique toxicity mechanism^70^.

**Table 4:** HPI values of the investigated HMs in the Nile River water.

Season	Site	Drinking ^43^		Drinking^44^		Irrigation ^45^		Aquatic life ^30^	
		HPI	Classification	HPI	Classification	HPI	Classification	HPI	Classification
Summer	S1	6300.88	Extreme Contamination	2074.23	Extreme Contamination	72.33	No Contamination	1571.49	Extreme Contamination
	S2	6433.23	Extreme Contamination	2070.04	Extreme Contamination	54.68	No Contamination	1345.05	Extreme Contamination
	S3	4848.22	Extreme Contamination	1521.80	Extreme Contamination	27.14	No Contamination	866.18	Extreme Contamination
	S4	1595.60	Extreme Contamination	580.30	Extreme Contamination	44.76	No Contamination	724.89	Extreme Contamination
Autumn	S1	3601.60	Extreme Contamination	1188.60	Extreme Contamination	44.94	No Contamination	934.92	Extreme Contamination
	S2	3146.45	Extreme Contamination	1025.01	Extreme Contamination	32.37	No Contamination	780.04	Extreme Contamination
	S3	1661.79	Extreme Contamination	578.24	Extreme Contamination	36.37	No Contamination	666.25	Extreme Contamination
	S4	1430.17	Extreme Contamination	484.21	Extreme Contamination	25.79	No Contamination	535.10	Extreme Contamination
Winter	S1	2636.08	Extreme Contamination	896.70	Extreme Contamination	45.74	No Contamination	820.39	Extreme Contamination
	S2	2404.97	Extreme Contamination	804.20	Extreme Contamination	36.72	No Contamination	692.64	Extreme Contamination
	S3	2165.60	Extreme Contamination	686.67	Extreme Contamination	18.68	No Contamination	473.38	Extreme Contamination
	S4	1653.56	Extreme Contamination	553.31	Extreme Contamination	26.87	No Contamination	497.26	Extreme Contamination
Spring	S1	3246.17	Extreme Contamination	1124.59	Extreme Contamination	61.21	No Contamination	1035.47	Extreme Contamination
	S2	2262.98	Extreme Contamination	779.13	Extreme Contamination	40.72	No Contamination	773.59	Extreme Contamination
	S3	2032.40	Extreme Contamination	688.25	Extreme Contamination	33.45	No Contamination	663.63	Extreme Contamination
	S4	1726.85	Extreme Contamination	572.77	Extreme Contamination	23.90	No Contamination	518.13	Extreme Contamination

### Bioaccumulation factor (BAF)

**Fig. 5:**
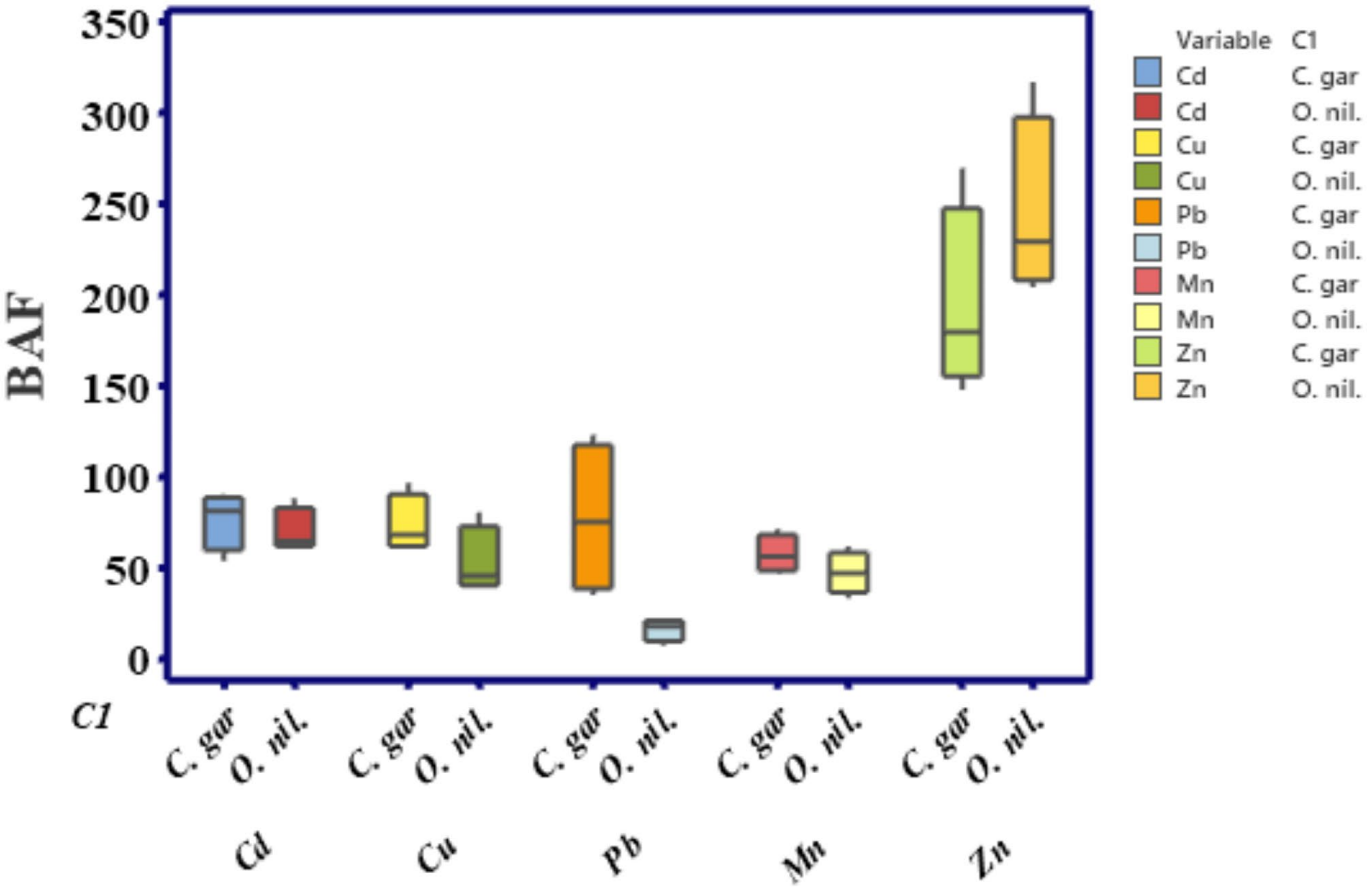
Boxplot of BAF values of Cd, Cu, Pb, Mn and Zn in the edible part of *O. niloticus* and *C. gariepinus*.

Fish might bioaccumulate HMs directly by consuming water and the food chain or from the sediment particles via the digestive system across biological membranes, including gills and muscles. Bioaccumulation of HMs in the different fish organs is greatly interspecific^72^. The BAF values of Cd, Cu, Pb, Mn, and Zn in the edible part varied within 61.33 - 88, 39.41–80.18, 6.82–21.99, 33.15–61.66, and 204-316.89, respectively, for *O. niloticus* and within 53.33-90, 60.95–96.3, 34.59-122.71, 46.44–71.14, and 147.51-269.44, respectively, for *C. gariepinus*, Fig. 5. No significant variations between the BAF measurements of all investigated HMs between the two fish species (unpaired t-test). BAF values of the investigated HMs were lower than 1000, so they indicated no probability of accumulation based on Olayinka–Olagunju et al. classification^73^. The BAF of the investigated HMs followed the order of Zn > Mn > Cd > Cu > Pb in *O. niloticus* and Zn > Pb > Cd > Cu > Mn in *C. gariepinus*. The variance in the accumulation levels of various fish species can primarily be ascribed to variances in the regulatory ability, behavior, and feeding habits^74^. In comparison, this accumulation pattern was slightly different from the pattern reported in *O. niloticus* muscles (Zn > Mn > Cu > Pb > Cd) in Great Cairo sector and the Nile rayahs discussed by Ghannam et al.^65^, and Taleb et al.^75^.

**Table 5:** Comparison of the HMs results (mg/g ww) in the muscles of *O. niloticus* and *C. gariepinus* with other species in different areas and the international standard permissible limits.

Study Area	Fish Sp.	Cd	Cu	Pb	Mn	Zn	Ref.
Great Cairo, Nile River	*Oreochromis niloticus*	0.2–0.28	2.01–5.41	0.21–1.11	12.1–15.25	20.91–32.52	Present Study
	*C. gariepinus*	0.2–0.35	3.12–6.5	1.52–3.62	15.01–17.72	15.12–26.92	
Great Cairo, Nile River	*Oreochromis niloticus*		1.62–7.71	0.02–0.26	0.2–1.89	4.93–47.17	68
	*S. galilaeus*		2.55–8.34	0.03–1.37	0.29–2.28	6.66–53.71	
Damietta branch, Nile River	*Oreochromis niloticus*	2.6–14.7	7.8–25.3	–	20.8–75.2	25.85–67.5	37
Rayahs, Nile River	*Nile tilapia*	Nd-2	Nd-18.6	Nd-15.4	1.1–56.90	8.4–98.4	65
	*Mango tilapia*	Nd-0.3	Nd-26.2	Nd-19.4	Nd-35.16	Nd-64.4	
	*Tilapia Zillii*	Nd-1.1	Nd-26.4	Nd-16.3	Nd-41	4.7–96.29	
El Bahr El pharaony	*Oreochromis niloticus*	0.021–0.037	0.82–3.65	1.72–5.64	0.24–2.52	5.35–16.56	42
North and Southeast Rivers, Iran	*Oncirhynchus mykiss trout*	–	5.8–8.4	3.2–6.6	–	6.2–11.2	13
Standard Permissible level			30	0.5		30	66
		0.5	30	0.5		40	64
			30		1	100	68
		0.2		2			60
		0.2	20	2		50	62
		0.1	10	0.1			61
		0.05		0.2			63
					20		69

### Daily intake (DI)

**Fig. 6:**
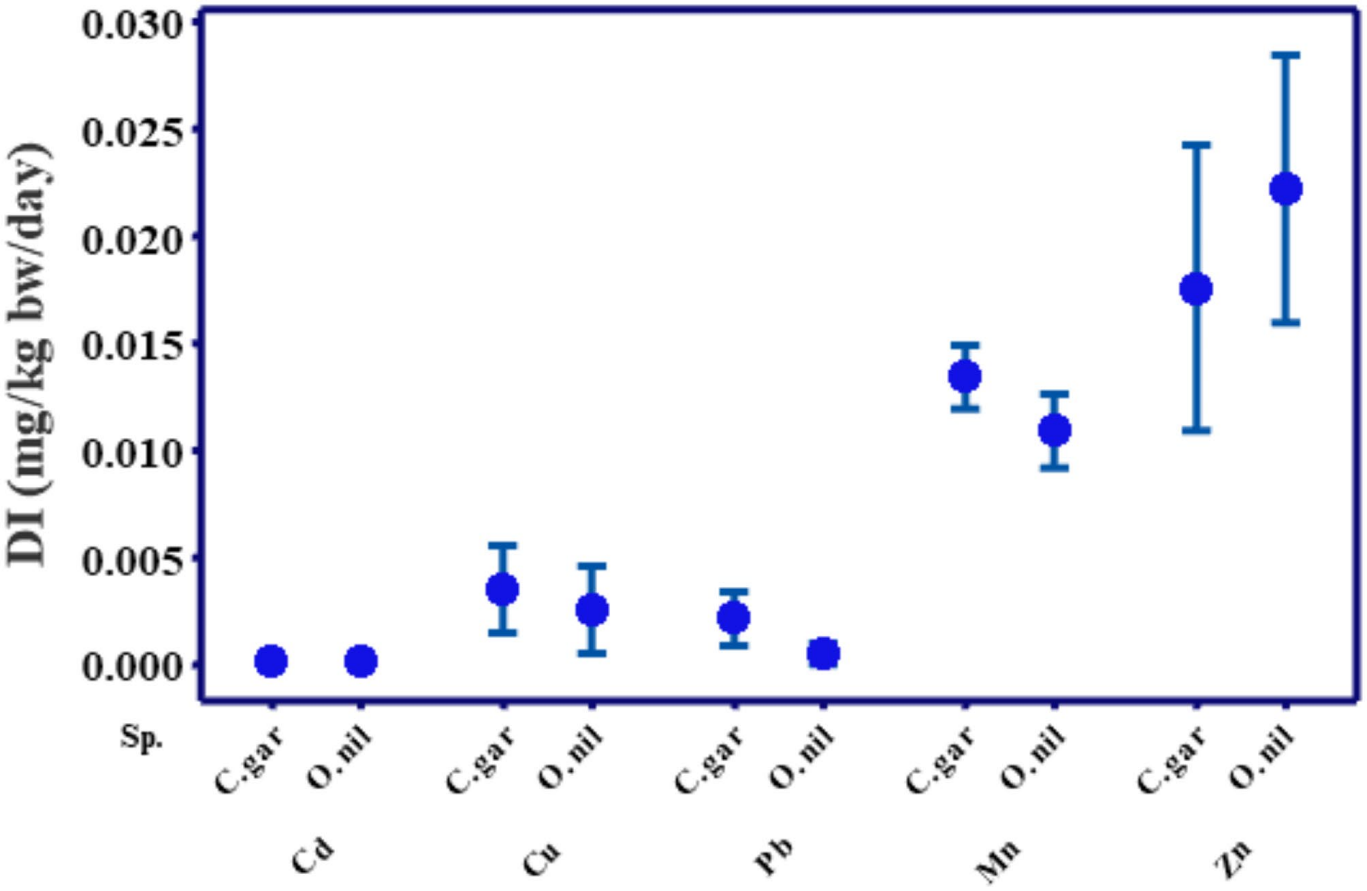
DI values of HMs by the intake of *O. niloticus* and *C. gariepinus* muscles collected from Great Cairo, Nile River.

Fish, positioned at the apex of the food structure, are the main target for the biomagnification of HMs and likely serve as channels for transfer to humans^76^. *O. niloticus* and *C. gariepinus* are the most common commercial fish species for human consumption in the Great Cairo Sector. Based on the consumption of 57 g of fish tissues by a 70 kg Egyptian person per day, the annual DI of Cd, Cu, Pb, Mn, and Zn from *O. niloticus* consumption was 0.0222, 0.0026, 0.0109, 0.0002, and 0.0005, mg/kg bw/day, respectively. Meanwhile, the DI.

of Cd, Cu, Pb, Mn, and Zn from *C. gariepinus* consumption was 0.0176, 0.0035, 0.0134, 0.0002, and 0.0022 mg/kg bw/day, respectively, Table 6. Moreover, the total DI values of all investigated HMs for *O. niloticus* were estimated to be 0.044, 0.037, 0.029, and 0.035 mg/kg bw/day during summer, autumn, winter, and spring, respectively. Meanwhile, they were; 0.044, 0.040, 0.032, and 0.033 mg/kg bw/day for *C. gariepinus* during summer, autumn, winter, and spring, respectively. Among the investigated HMs, Zn, and Mn constituted the highest estimated DI than Cu, Pb, and Cd (Fig. 6).

**Table 6:** The DI (mg/kg bw/day), THQ, and HI values of HMs from the consumption of *O. niloticus* and *C. gariepinus* muscles collected from the Great Cairo sector, the Nile River.

	*O. niloticus*				*C. gariepinus*			
DI (mg/kg bw/day)	Summer	Autumn	Winter	Spring	Summer	Autumn	Winter	Spring
Cd	0.00023	0.00016	0.00018	0.00019	0.00029	0.00022	0.00017	0.00016
Cu	0.00441	0.00252	0.00164	0.00173	0.00529	0.00368	0.00265	0.00254
Pb	0.00090	0.00059	0.00042	0.00017	0.00181	0.00269	0.00295	0.00124
Mn	0.01242	0.01084	0.00985	0.01059	0.01443	0.01328	0.01380	0.01222
Zn	0.02647	0.02322	0.01703	0.02216	0.02192	0.01975	0.01231	0.01646
$\:\sum\:DI$ (mg/kg bw/day)	0.044	0.037	0.029	0.035	0.044	0.040	0.032	0.033
THQ	Summer	Autumn	Winter	Spring	Summer	Autumn	Winter	Spring
Cd	0.23	0.16	0.18	0.19	0.29	0.22	0.17	0.16
Cu	0.11	0.06	0.04	0.04	0.13	0.09	0.07	0.06
Pb	0.26	0.17	0.12	0.05	0.52	0.77	0.84	0.35
Mn	0.09	0.08	0.07	0.08	0.10	0.09	0.10	0.09
Zn	0.09	0.08	0.06	0.07	0.07	0.07	0.04	0.05
HI	0.77	0.55	0.47	0.43	1.11	1.24	1.22	0.72

### Target hazard quotient and hazard index

**Fig. 7:**
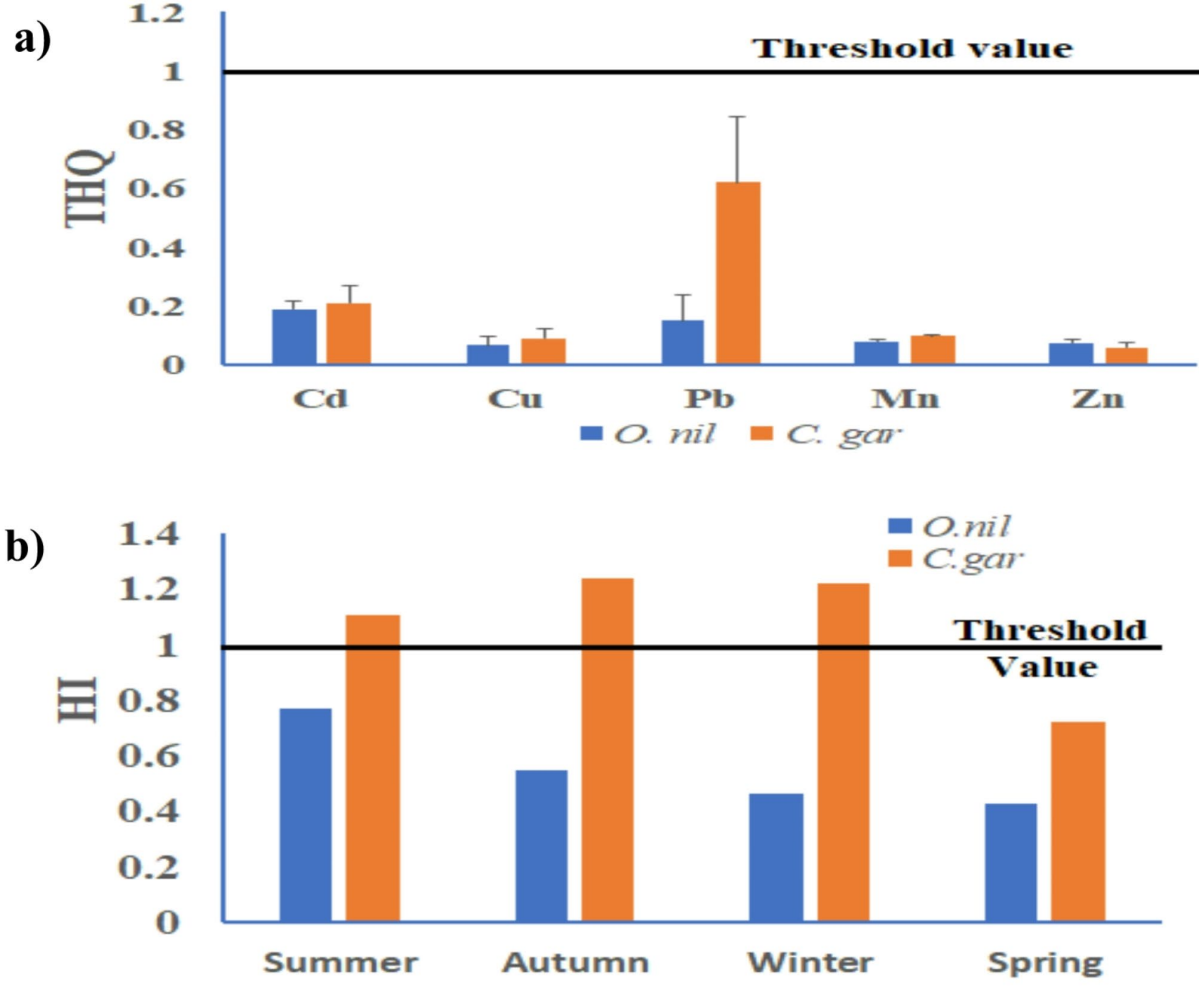
(**a**) THQ values and (**b**) HI of HMs in muscles of *O. niloticus* and *C. gariepinus* collected from the Great Cairo Sector, Nile River.

The THQ values explained the single effect of each HM by the fish tissues´ intake. The present results explored an absence of non-carcinogenic risk of all the HMs in the two fish species with THQ values < 1, Fig. 7a. They were in the order of Cd (0.189) > Pb (0.148) > Mn (0.078) > Zn (0.074) > Cu (0.064) for *O. niloticus* and in the order of Pb (0.620) > Cd (0.210) > Mn (0.096) > Cu (0.089) > Zn (0.059) for *C. gariepinus.* Following Custodio et al.^77^, eating either if the two fish species does not present any non-carcinogenic health hazards when consuming the examined HMs separately. The annual mean HI value was estimated to be 0.55 ± 0.15 and 1.07 ± 0.24, for *O. niloticus* and *C. gariepinus*, respectively. The HI value was found to be lower than the permitted level of 1 in the muscles of *O. niloticus* in all seasons, (Fig. 7b). Thus, they referred to no health risk to consumers from *O. niloticus* consumption in the study area. On the other hand, HI values of *C. gariepinus* slightly exceeded the threshold value of 1 during summer, autumn, and spring, classified as moderate risk levels for consumers. Pb and Cd were the major contributing metals in HI where Pb contributed 60.71% HI, and Cd with 17.94%, followed by Mn (8.85%), then Cu (7.29%), and Zn (5.18%). The HI results referred to serious problems to human health by the intake of *C. gariepinus* fishes from the Great Cairo Sector. The findings of the research highlight the urgent need for enhanced environmental management strategies to mitigate heavy metal contamination in the Great Cairo Sector. The estimated HI value in both *O. niloticus* and *C. gariepinus* was lower than that reported for the crayfish (1.27–1.92), and (22.9–3.22) collected from El-Kanater Station and El-Rahawy drain, respectively^35^. In comparison, El-Haddad et al.,^68^ reported no carcinogenic risk (HI < 1) of consuming *O. niloticus* or *S. galilaeus* from the Nile River Islands. Additionally, the results of the health risk assessment justified the importance of monitoring and regulating heavy metal levels in fish species to protect the health of consumers.

## Materials and methods

### Study area

**Fig. 8:**
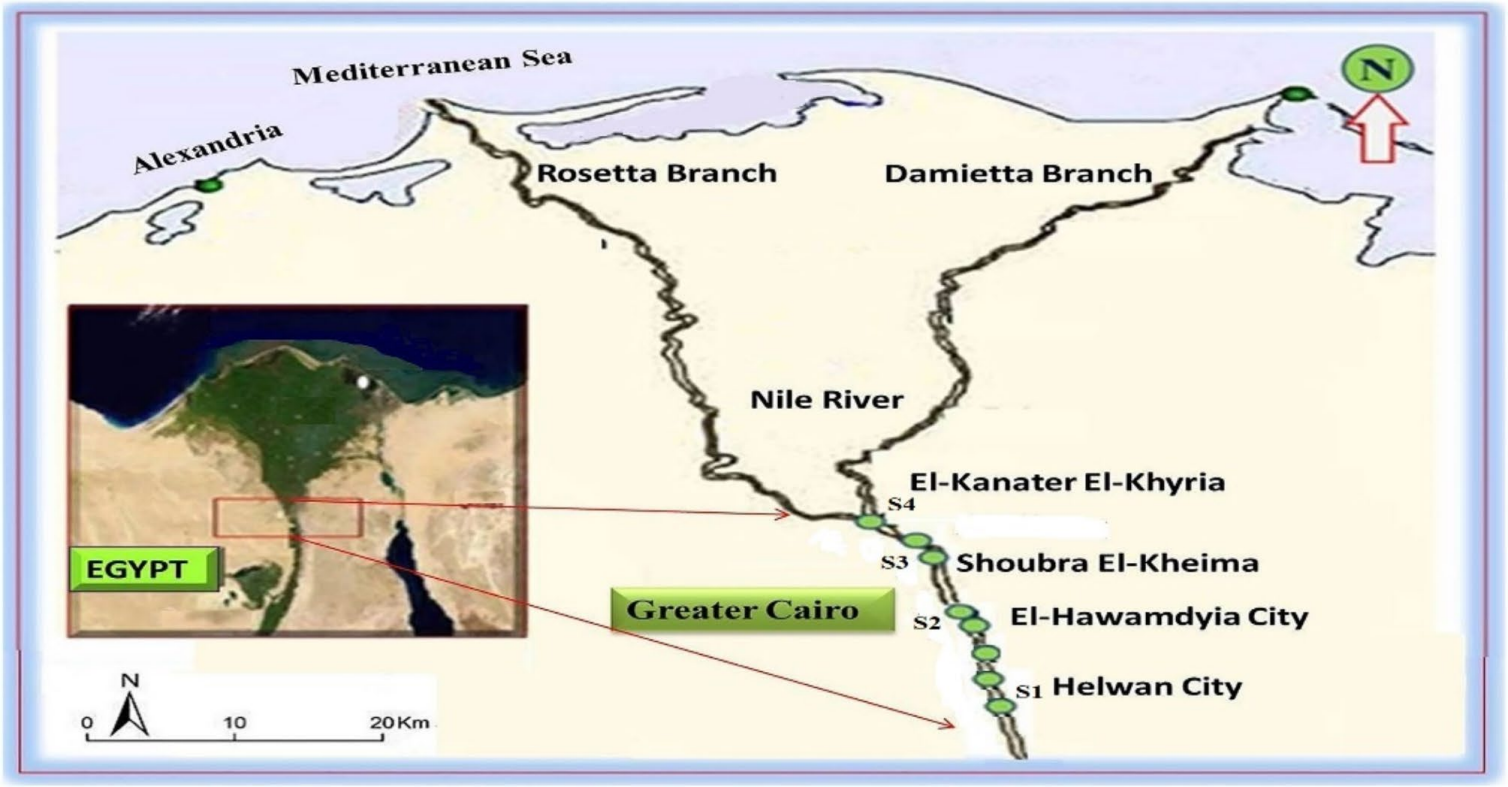
Map showing sampling sites of the Great Cairo, Nile River^54^.

The Nile River is a prominent characteristic of Africa’s northeastern basin. It is a significant northward-flowing river in northeastern Africa that arises in Ethiopia and empties into the Mediterranean Sea^75^. The Nile is the largest river in Africa and has traditionally been regarded as the longest river globally, extending around 6,825 km. The Nile serves as the principal water source for Egypt, Sudan, and South Sudan. The Nile River in Egypt is subjected to various pollutants from both point and non-point sources along its coasts^78^. Numerous pollutant categories from diverse sources, including sewage, residential, industrial, and agricultural waste effluents, are dumped into the Nile from El-Rahawy drains, exceeding 5 × 10^8^ m³ per day^79^. In the Delta region, the Nile bifurcates into two primary branches (Rosetta and Damietta) and four subsidiary branches (irrigation canals, referred to as rayahs in colloquial dialect)^80^. This article pertains to four locations within the Great Cairo Sector of the Nile River. They were chosen based on their proximity to certain polluting activities, (Fig. 8). Table (7) presents the locations and descriptions of sites, together with the types of human activity conducted there.

### Sample collection and preservation

#### Water

Forty-eight surface water samples (0–0.5 cm depth) were obtained from four sites along the Nile River, spanning from Helwan to El-Qanater El-Khayria, between summer 2021 and spring 2022. The sampling sites (S1–S3) were selected due to their closeness to anticipated emission sources, while S4 was considered as a control as it is far from direct pollution sources (Table 7). Prior to water collection, the sampling bottles were washed with distilled water and eventually soaked in 10% HNO_3_. The samples were collected as triplicates from each site. Water samples were obtained using a 2 L polyvinyl chloride Van Dorn bottle water sampler, stored in an ice box, and then transported to the laboratory. For HMs analysis, the collected samples were acidified with 0.5% HNO_3_ in well-labeled bottles and were stored at room temperature (4 °C) prior to laboratory evaluation.

### Fish

Two fish species (*Oreochromis niloticus* and *Clarias gariepinus*) were collected seasonally using a fisherman’s net. These two species are the most commonly species in the Nile River and the highest consumption by humans. Nearly 30 adult samples from each species were considered and analyzed in triplicates. we collected *O. niloticus* during summer (mean length 15.9 ± 5.2 cm and mean weight 91.8 ± 5.1 g), autumn (mean length 15.8 ± 1.1 cm and mean weight 91.1 ± 7.1), winter (mean length 17.3 ± 2.2 cm and mean weight 95.6 ± 3.8), and spring (mean length 17.9 ± 5.1 cm and mean weight 97.6 ± 3.7) and *C. gariepinus* (mean length 25.3 ± 2.7 cm and mean weight 125.5 ± 3.2 g) during summer, (mean length 23.4 ± 4.1 cm and mean weight 122.9 ± 5.3) during autumn, (mean length 26.4 ± 5.4 cm and mean weight 132.6 ± 5.2) during winter and (mean length 24.4 ± 4.3 cm and mean weight 127.6 ± 2.8 g) during spring from the great Cairo sector. The fish samples were securely contained in plastic bags and brought immediately to the laboratory in an icebox at 4 °C, where they were subsequently sliced to extract the muscle tissue.

**Table 7:** Locations and features of the sampling sites.

Site	Feature	Activities	Latitude	Longitude
S1	Helwan, In Front of Iron and Steel company	I, D	29°48′0″N	31°17′45″E
S2	El-Hawamdiya, in front of Starch and Glucose Company	I, D	29°52′31″N	31°17′3″E
S3	Shobra, In front of the Electrical power Station	I, D, S	30° 7′29″N	31°14′4″ E
S4	El-Qanater El-Khayria, In front of the water purification Station	A	30°11′1″N	31° 8′20″E

Where A: Agricultural, I: Industrial, D: Domestic activities.

### Analysis

#### Physicochemical parameters

The water samples’ temperature, pH, electrical conductivity (EC), and dissolved oxygen (DO) levels were promptly evaluated using a Thermo Orion Star (A 329 multiparameter analyzer) during sample collection. The analytical methodologies are addressed in the American Public Health Association^81^. The biological oxygen demand (BOD) was assessed using the 5-day incubation method outlined in APHA^81^. The potassium permanganate method was employed to determine chemical oxygen demand (COD)^81^.

### Heavy metals in water

Water samples were subjected to digestion utilizing the Nitric Acid Digestion Method outlined in APHA^81^. Approximately 5 ml of concentrated HNO_3_ was introduced to a 250 ml water sample, which was subsequently heated on a hot surface until reduced to 50 ml volume without inducing precipitation. The levels of heavy metals were quantified using Eq. (1):

1 $$HMs~\left( {\mu g/L} \right)=\frac{{{C_i} \times {V_f}}}{{{V_i}}}$$

where C_i_ represents the ICP reported metal concentration in the digested solution (µg/l), V_f_ denotes the final volume of the digested solution (ml), and V_i_ indicates the initial water sample volume (ml).

### HMs in fish muscles

The heavy metals in fish muscles were digested following the protocol outlined by FAO/SIDA^82^. A 10 ml aliquot of a freshly produced mixture of Concentrated nitric acid (65%) and perchloric acids (70%) (HNO_3_: HClO_4_ = 1:1) was introduced to 5 g of muscle tissue from each species in sealed Teflon containers. The samples were digested for 30 min at 160 °C in a microwave oven (model Milestone, MLS-1200 mega, Germany) until a transparent solution was obtained. The extract was adjusted with double distilled water up to a 50 ml solution in a calibrated measuring flask (50 ml), subsequently filtered, and put into plastic vials for analysis. Blanks were established in each digesting protocol. The amounts of heavy metals in fish muscle tissue were determined using Eq. (2):

2 $$HMs~\left( {\mu \frac{g}{g}wet~wt} \right)=\frac{{{C_i} \times {V_f}}}{{1000 \times {W_t}}}~$$

where C_i_ represents the HMs content in the digested solution (µg/l), V_f_ denotes the final volume of the digested solution (ml), W_t_ is the weight of the fish muscle sample (g), and 1000 is the unit conversion factor.

### Instrument operation, calibration, and quality control

The total levels of the HMs (Cd, Cu, Pb, Mn, and Zn) in the digested water and fish samples were quantified using Inductively Coupled Plasma Emission Spectrometry (ICP-ES) with Ultra Sonic Nebulizer (USN), model Perkin Elmer optima 7000, USA. Before being utilized, all glassware was cleansed with diluted HCl. The chemicals used were from Germany (E. Merck). A 1000 g/L multi-element validated standard solution served as the baseline stock for apparatus calibration. The standard solutions were prepared with filtered, double-distilled water. The HMs analysis was dependent on the calibration curves (CC), plotted at five different concentrations. Before conducting the sample analyses, runs were carried out for each HM to determine the correlation coefficient (r^2^) values on the CC. Furthermore, the HMs concentrations were verified utilizing certified Reference materials via the National Institute of Standards and Technology (NIST). The accuracy and precision of the procedure were verified through 3 replicate readings of the standard materials, yielding metal recovery rates between 97.1% and 103.8%, which are within the permitted recovery percent between 80% and 110% defined by Huber^83^. The wavelengths (nm) for ICP-ES are as follows: 226.499, 324.747, 220.35, 257.604, and 213.855 nm, with corresponding detection limits (µg/L) of 0.1, 0.3, 1.5, 0.04, and 0.2 µg/L, and quantification limits (µg/L) of 0.15, 0.4, 1.8, 0.1 and 0.3 for Cd, Cu, Pb, Mn, and Zn respectively. The operational parameters of the ICP instrument are detailed in Table S3 in the supplementary file.

### Metal quality indices (MQI) in water

This study employed many metal indices to evaluate the levels of metal contamination in the Nile River, relevant to drinking water, irrigation, and aquatic life applications. These indices included the Pollution Index (PI), Metal Index (MI), and Heavy Metal Pollution Index (HPI). The PI expresses the single effect of each individual HM by Eq. (3) with different grades reported in Table 8. Moreover, MI explains the combined effect of all investigated HMs. A greater concentration of a metal relative to its maximum permissible limit (MAC) indicates worse water quality (Eq. 4). The HPI elucidates metal pollution by considering the cumulative effects of different heavy metals, utilizing the weighted arithmetic mean method^41^ as outlined in Eq. (5) and Table (8).

### Bioaccumulation factor (BAF)

Bioaccumulation of HM may transpire through interaction with gills and skin and/or through intake of these metals in the digestive system^84^. The formula (Eq. 6) can be employed to identify the BAF in the consumable muscle of the species under consideration.

### Human health risk assessment

Individuals are subjected to HMs in three ways, i.e., inhaling, intake, and interactions through the skin^85^. The most common route is the ingestion of HMs through fish consumption. The average daily intake (DI) of each HM was estimated based on a diet of 57 g/day of fish tissues^87^. The target hazard quotient (THQ), a non-cancer estimation of adverse health effects linked to the ingesting of HMs pollutants accumulating in fish tissues, was employed to assess hazard risk^84^. Each HM´s THQ was established. Furthermore, the hazard index (HI) is a mathematical computation that accounts for the impact of non-carcinogenic risks by using the THQ values of all the HMs under investigation. The equations of DI, THQ, and guideline criteria are in Table 8.

**Table 8:** The equations and evaluation criteria of HMs pollution indices used in water, BAF, and human health risk indices of HMs in fish tissues.

No.	Equation	Variables	Evaluation criteria	References
Equation 3	$PI=\frac{{\sqrt {{{\left( {\frac{{{C_i}}}{{{S_i}}}} \right)}^2}_{{max}}+~{{\left( {\frac{{{C_i}}}{{{S_i}}}} \right)}^2}_{{min}}} }}{2}$	Where C_i_ signifies the HM concentration, S_i_ denotes the HM level outlined in the national water quality criteria.	PI < 1, no effect 2 > PI > 1, Slightly affected 3 > PI > 2, Moderately affected 5 > PI > 3, Strongly affected PI > 5, Seriously affected	^88^
Equation 4	$MI=\mathop \sum \limits_{{i=1}}^{n} \frac{{{C_i}}}{{MA{C_i}}}$	n is the total number of assessed HMs, MAC is the maximum permitted concentration of the i^th^ metal.	MI > 1, a critical threshold	^89,90^
Equation 5	$HPI=\frac{{\mathop \sum \nolimits_{{i=1}}^{n} {Q_i}{W_i}}}{{\mathop \sum \nolimits_{{i=1}}^{n} {W_i}}}$ ${W_i}=\frac{1}{{{S_i}}}$ ${Q_i}=\frac{{{C_i}}}{{{S_i}}} \times 100$	Q_i_ is the sub-index of the ith metal, n denotes the quantity of assessed HMs, and W_i_ is the weight unit of the ith metal (between 0 and 1).	HPI < 100, no contamination, HPI = 100, Moderate Contamination, HPI > 100, Extreme Contamination and unsuitable for drinking.	^41,91–93^
Equation 6	$BAF=\frac{{{M_t}}}{{{M_w}}}$	M_t_ is the metal concentration in fish tissues (µg/g wet weight) and M_w_ is the metal concentration in water (µg/l).	BAF < 1000, little possibility of accumulation. 5000 > BAF > 1000, an intermediate bioaccumulative metal. BAF > 5000, a highly bioaccumulative metal.	^73^
Equation 7	$DI=\frac{{{C_i} \times IFR \times EF \times ED}}{{BW \times A{T_n}}} \times ~{10^{ - 3}}$	C_i_ is the average concentration of HMs in fish (mg/kg dry weight), IFR is the fish ingestion rate (57 g/person/day), BW is the average adult body weight (70 kg), ED signifies the exposure period (70) years as average lifetime, EF refers to the exposure frequency (365 days/year). AT_n_ is the average exposure time for non-carcinogens (assuming 70 years).		^94^
Equation 8	$THQ=\frac{{DI}}{{RFD}}$	RFD is the oral reference dose, 0.001, 0.04, 0.0036, 0.14, and 0.3 for Cd, Cu, Pb, Mn, and Zn, respectively.	THQs < 1, No harmful effects are anticipated. THQs > 1, a potential threat to health exists.	^87^
Equation 9	$HI=\mathop \sum \limits_{{i=1}}^{n} TH{Q_s}$	N: number of measured HMs.	HI < 1, no anticipated harmful effects HI > 1, a potential health threat	^87^

### Statistical analysis

The current data were examined for variations in time and space utilizing Minitab 16^®^ Statistical Software (Minitab Inc.), with statistical significance established at *p* < 0.05 and very significant findings at *p* < 0.01. Anderson Darling tests were performed to verify the assumptions of parametric tests, and the results indicated that the variables conformed to a normal distribution pattern. A correlogram was generated utilizing R-Studio version 2022.07.1 (R-Studio, Boston, MA, USA). The Pearson correlation coefficient (r) for the measured variables was analyzed using the R program, with code provided in the supplementary file. Triplicate analyses of heavy metals in fish tissues were performed, and the results were presented as mean ± standard deviation. The one-way analysis of variance (ANOVA) and Tukey comparison test were used to ascertain any statistically significant differences in the means of the physicochemical characteristics and heavy metal concentrations at a 5% confidence level across various seasons and locations. Unpaired t-tests were used to compare the HM levels and bioaccumulation between the two fish species.

## Conclusion

The proliferation of HMs contamination in the aquatic environment has escalated. Their anthropogenic sources include industrial emissions, domestic, and farming techniques, including the application of fertilizers and pesticides. Global monitoring of drinking water sources is essential. This study evaluated the seasonal and regional distribution of some HMs (Cd, Cu, Pb, Mn, and Zn) in the Great Cairo sector, Nile River, Egypt over the study period (2021–2022). Forty eight surface water samples were collected from the study area and analyzed for physicochemical parameters and HMs content. Furthermore, this study investigated two fish species (*O. niloticus and C. gariepinus)* to evaluate the HMs distribution, bioaccumulation and heath risk implications. The HMs results in water decreased in the rank; Mn > Zn > Pb > Cu > Cd. The pollution index results indicated that essential Cu and Zn had a negligible pollution effect at all sites under investigation according to WHO drinking water criteria. Moreover, Mn showed a moderate PI effect. On the other hand, the nonessential Pb was seriously affected at all sites while Cd has a slight pollution effect only at S1 while negligible effects at the other sites. The metal index and heavy metal pollution index results outline that HMs exceeded the permissible levels for drinking water and aquatic life utilization and can induce both chronic and acute toxicity and then threaten the human health. Accordingly, obligatory measures must be implemented, and remediation strategies must be advised. The concentrations of HMs in the investigated species ranked in the order: (Zn > Mn > Cu > Pb > Cd), with Cd and Pb exceeding their respective permissible limits. In addition, no significant variations between the BAF measurements of all investigated HMs between the two fish species (unpaired t-test). The Hazard Index results indicated that the HMs in *O. niloticus* do not present any specific concerns regarding individual health issues and are safe for human consumption. Conversely, the HI in *C. gariepinus* surpasses the acceptable threshold, and thus an intensive evaluation must be implemented towards this species. This study recommends constant monitoring of industrial effluents for conducting different effective strategies to eliminate metal ions. Furthermore, a multitude of regulations and legislation must be established for the conservation of water resources. In addition, this work can inform policymakers and stakeholders about the potential consequences associated with HM contamination in aquatic systems, facilitating the creation of tailored policies and laws to mitigate these issues.

## Electronic supplementary material

Below is the link to the electronic supplementary material.

Supplementary Material 1

## Data Availability

All data generated or analyzed during this study are included in this published article [and its supplementary information files].
