# Supplementary material for: Evaluation of some heavy metals in water and health implications for fish consumers of the Great Cairo Sector of the Nile River
Source: Sci Rep. 2025 Apr 12;15:12632. doi: 10.1038/s41598-025-95308-z (PMC11993652; doi:10.1038/s41598-025-95308-z)
Supplement: Supplementary file 1 — Supplementary Material 1 [file 41598_2025_95308_MOESM1_ESM.docx]

**Supplementary file**

**Evaluation of some heavy metals in water and Health Implications for Fish Consumers of the Nile River**

**Alaa I. Khedr ^1,^ * & Hala E. Ghannam ^2^**

^1^ Chemistry Laboratory, National Institute of Oceanography and Fisheries, NIOF, Cairo, Egypt.

^2^ Pollution Laboratory, National Institute of Oceanography and Fisheries, NIOF, Cairo, Egypt.

*Corresponding Author, E-mail: [alaaibrahem40@yahoo.com](mailto:alaaibrahem40@yahoo.com), ORCID ID 0000-0001-8630-8441.

**Results and Discussion**

**Table S1: physicochemical variables (Average ± SD) in in the Great Cairo Sector, Nile River, Egypt.**

| **Season** | **Site** | **Temp. (ºC)** | **pH** | **EC (µs/cm)** | **DO (mg/l)** | **COD (mg/l)** | **BOD (mg/l)** |
| --- | --- | --- | --- | --- | --- | --- | --- |
| **Summer** | **S1** | 30.7±0.15 | 7.75±0.2 | 511±4.58 | 6.63±0.17 | 11.2±0.25 | 4.7±0.25 |
| **Summer** | **S2** | 30.4±0.2 | 7.77±0.19 | 413±9.29 | 6.375±0.2 | 14.4±0.5 | 4.9±0.35 |
| **Summer** | **S3** | 31.7±0.2 | 7.79±0.21 | 415±4.04 | 6.3±0.32 | 16.4±0.15 | 6.5±0.35 |
| **Summer** | **S4** | 30±0.25 | 7.81±0.2 | 420±3.51 | 6.04±0.2 | 14.2±0.45 | 4.02±0.17 |
| **Autumn** | **S1** | 24.1±0.08 | 7.45±0.15 | 332±3.46 | 6.885±0.3 | 7.1±0.15 | 4.3±0.25 |
| **Autumn** | **S2** | 25.7±0.51 | 7.34±0.1 | 340±4.58 | 7.395±0.13 | 10.2±0.46 | 4.5±0.25 |
| **Autumn** | **S3** | 26.9±0.12 | 7.23±0.15 | 239±1.53 | 6.5±0.4 | 11.9±0.4 | 5.2±0.4 |
| **Autumn** | **S4** | 24.6±0.42 | 7.12±0.06 | 271±6.34 | 6.29±0.09 | 5.2±0.93 | 4±0.17 |
| **Winter** | **S1** | 17.5±0.4 | 8.6±0.21 | 378±4.93 | 7.3±0.25 | 7±0.25 | 4.4±0.25 |
| **Winter** | **S2** | 16.6±0.46 | 8.81±0.1 | 382±3.61 | 7.9±0.26 | 9.5±0.5 | 3.8±0.21 |
| **Winter** | **S3** | 19.2±0.53 | 9.02±0.01 | 379±4.16 | 8±0.15 | 9.5±0.15 | 4.3±0.17 |
| **Winter** | **S4** | 16.3±0.25 | 9.23±0.16 | 333±2.52 | 8.5±0.32 | 7.1±0.12 | 3.3±0.44 |
| **Spring** | **S1** | 20.9±0.55 | 7.95±0.16 | 642±2 | 6.715±0.04 | 10.2±0.15 | 5.3±0.25 |
| **Spring** | **S2** | 20.2±0.25 | 7.97±0.07 | 551±3.79 | 6.375±0.22 | 11.1±0.15 | 4.5±0.4 |
| **Spring** | **S3** | 22.3±0.21 | 7.99±0.03 | 429±5.57 | 6.46±0.29 | 9.3±0.25 | 5.5±0.5 |
| **Spring** | **S4** | 20.1±0.26 | 8.01±0.02 | 453±4.04 | 7.055±0.2 | 8.5±0.36 | 5.1±0.2 |

**Table S2:** Heavy Metals concentrations (Average ± SD) µg/l in in the Great Cairo Sector, Nile River, Egypt.

| **Season** | **Site** | **Cd (µg/l)** | **Cu (µg/l)** | **Pb(µg/l)** | **Mn(µg/l)** | **Zn(µg/l)** |
| --- | --- | --- | --- | --- | --- | --- |
| **Summer** | **S1** | 7±0.4 | 85.5±1.04 | 84±3.51 | 316.00±4.0 | 155±6.03 |
| **Summer** | **S2** | 5±0.25 | 67.5±2.02 | 86±2.65 | 352.00±6.56 | 170±9.5 |
| **Summer** | **S3** | 2±0.15 | 56.25±4.0 | 65±4.04 | 344±4.51 | 160±14.42 |
| **Summer** | **S4** | 4±0.31 | 60.75±5.5 | 21±1.53 | 320.00±5.57 | 110±4.58 |
| **Autumn** | **S1** | 4±0.21 | 60.75±0.88 | 48±2.08 | 328.00±6.03 | 110±2.52 |
| **Autumn** | **S2** | 3±0.25 | 56.25±4.5 | 42±1.54 | 176.00±6.0 | 100±2.08 |
| **Autumn** | **S3** | 3±0.21 | 69.75±2.56 | 22±1.53 | 340±2.52 | 85±2.52 |
| **Autumn** | **S4** | 2±0.16 | 65.25±3.5 | 19±1 | 276.00±2.52 | 65±2.65 |
| **Winter** | **S1** | 4±0.17 | 56.25±3.4 | 35±2.52 | 368.00±2.0 | 125±2.52 |
| **Winter** | **S2** | 3±0.12 | 52.75±2.5 | 32±3.61 | 372.00±2.52 | 115±2.65 |
| **Winter** | **S3** | 1±0.15 | 50±3.06 | 29±2.08 | 376.00±3.61 | 100±2.08 |
| **Winter** | **S4** | 2±0.04 | 45±3.0 | 22±1.53 | 344.00±3.06 | 70±4.58 |
| **Spring** | **S1** | 6±0.25 | 45±3.51 | 43±3 | 264.00±5.03 | 130±11.5 |
| **Spring** | **S2** | 4±0.2 | 54±3.0 | 30±2.52 | 148.00±3.0 | 125±3.51 |
| **Spring** | **S3** | 3±0.12 | 56.25±2.05 | 27±2.52 | 224.00±5.0 | 105±3.51 |
| **Spring** | **S4** | 2±0.13 | 49.5±2.5 | 23±2.08 | 208.00±2.08 | 95±4.51 |

**Material and Methods**

The code utilized in doing the correlogram on R studio is

Corrplot (cor(data), method= "circle", type = "lower", number.cex = 0.7, number. font = 1, cl.cex =0.8, add Coef = TRUE).

**Table S3**: ICP-OES operating conditions for each analyzed metal.
